# Is There a Contribution of Structural Brain Phenotypes to the Variance in Resting Energy Expenditure before and after Weight Loss in Overweight Females?

**DOI:** 10.3390/nu11112759

**Published:** 2019-11-14

**Authors:** Corinna Geisler, Manfred J. Müller

**Affiliations:** 1 Institute of Human Nutrition and Food Science, Christian-Albrechts-University of Kiel, Düsternbrooker Weg 17-19, D-24105 Kiel, Germany; mmueller@nutrfoodsc.uni-kiel.de; 2 Division of Endocrinology, Diabetology and Clinical Nutrition, Department of Internal Medicine 1, University Hospital Schleswig-Holstein, Campus Kiel, University of Kiel, Düsternbrooker Weg 17-19, D-24105 Kiel, Germany

**Keywords:** weight loss, resting energy expenditure, brain, gray matter, white matter

## Abstract

Brain gray (GM) and white matter (WM) are associated with resting energy expenditure (REE). The impact of weight loss on GM and WM masses, as well as on their associations with REE and the ratio between body and brain metabolism, i.e., encephalic measure (EM)), are unknown. Longitudinal data of 69 female Caucasian subjects (age range 19–69 years) with detailed information on fat mass (FM), fat free mas (FFM), GM, WM and REE. Mean weight loss was 14.5 ± 11.9 kg with changes in FM (−12.9 ± 9.8 kg), FFM (−1.7 ± 4.8 kg) and REE (−159 ± 191 kcal/24 h) (all *p* < 0.05). With weight loss, there were no changes in GM and WM. Before and after weight loss, FFM was the main determinant of REE (*r*^2^ = 0.483 and 0.413; *p* < 0.05). After weight loss, GM added to the variances in REE (3.6%), REE_adjFFM_ (6.1%) and the REE on FFM residuals (6.6%). In addition, before and after weight loss GM explained 25.0% and 10.0% of the variances in EM (*p* < 0.05). Weight loss had no effect on volumes of GM and WM. After weight loss, both, GM added to the variances of REE, REE on FFM residuals and EM.

## 1. Introduction

Although brain mass accounted for 2% of one’s total bodyweight, the metabolic rate of the human brain contributes to 20% of resting energy expenditure (REE) [1,2]. The brain has a specific metabolic rate of about 240 kcal/kg, which is 18-fold higher than that of skeletal muscle (i.e., 13 kcal/kg). Morphologically, brain mass has two main tissue structures, gray matter (GM) and white matter (WM). GM and WM differ in their metabolic rates, i.e., the energy need of GM is 163 kcal/kg compared to 76 kcal/kg for WM.) [3]. We speculated that besides brain mass, brain tissue composition adds to the inter-individual variance in REE. This idea was supported by our previous cross-sectional data [4], where the fat free mass (FFM) explained 70.6% of the variance in REE, and GM explained a further 5.6%.

The allocation of energy between body and brain is essential for brain function and control of energy balance. This has been described as an encephalic measure (EM) [5,6]. At a high EM a greater part of whole body energy is allocated to the brain. EM was lower in subjects with obesity than normal weight subjects which was improved with weight loss, while the relative energy consumption of the brain is decreased in subjects with obesity [5,6]. These data are evidence that ‘body-brain energy allocation’ changes with weight loss. 

With weight loss, the brain is the only organ that does not lose mass [5]. However, weight loss may affect brain structures in obese subjects [7]. In fact, in morbidly obese patients, a considerable weight loss after bariatric surgery improved WM, as well as GM densities [8,9]. Accordingly, a six to 12 week very low calorie diet resulted in a weight loss of about 10 kg resulted in reduced WM with unchanged [10] or increased GM [11]. However, the impact of obesity on brain structures is controversial, e.g., there were both, positive and negative correlations between BMI and the volumes of GM [12]. In addition, a high BMI was related to low WM in subcortical and cortical regions of the brain [13,14]. By contrast, other authors showed no [15] or a positive [16,17] relationship between BMI and WM. 

The present study adds to our previous work [4] with a specific focus on the effect of weight loss on GM, WM and their associations with REE. We have used magnetic resonance imaging (MRI) to investigate the influence of weight loss on brain compartments. Specifically, we have addressed two research questions. First, is there an influence of weight loss on GM and WM? Second, do GM and WM affect the REE on FFM association, as well as EM before and after weight loss?

## 2. Materials and Methods 

This is a secondary data analysis of data obtained in the “Reference Center for Body Composition” (Institute of Human Nutrition and Food Science of the Christian-Albrechts University Kiel, Germany) [2,18,19,20,21,22]. For analysis 69 female subjects were selected, mean age of 36 ± 9.8 years (range, 19.4 to 68.8 years), a BMI of 38.9 ± 7.5 kg/m^2^ (range, 28.2 to 58.7 kg/m^2^), the mean follow-up time was 17.9 ± 7.1 weeks (range, 9 to 35 weeks). The subjects had participated in different weight loss studies which had been published previously; 43 subjects with a six months low-caloric diet [23,24], nine subjects during controlled underfeeding for three weeks [18,20] and 17 subjects with bariatric surgery [21].

### 2.1. Details of Study Populations

#### 2.1.1. Study Group 1

The original study population was recruited between February 2010 and September 2012. During the study period, 32 subjects were observed. The detailed study protocol and corresponding results were published earlier [18,20]. Subjects were participants of a controlled intervention study and consumed a low-caloric diet. During the low-caloric diet, daily energy requirements were reduced by 50%. A follow-up period was three weeks. Out of the 32 subjects, nine subjects with MRI images at baseline and follow-up were included in the present data analysis (aged 23.9–40.2 years; mean BMI 41.05 kg/m^2^). The trial was registered at clinical trials.gov as NCT01737034.

#### 2.1.2. Study Group 2

During 2006 and 2009 were 96 subjects with overweight recruited and observed. Subjects were included in a six-month intervention study. Study details were described elsewhere [23,24]. Out of 96 subjects, 43 subjects with MRI images at baseline and follow-up (six months) were included in brain data analysis (aged 19.4–42.6 years; mean BMI 34 kg/m^2^).

#### 2.1.3. Study Group 3

In the study group, three 32 extremely obese subjects were included and recruited during 2009 and 2010. All subjects had bariatric surgery with a follow-up time of six-month [21]. Out of 32 subjects, 17 subjects with MRI images at baseline and follow-up were included in brain data analysis (aged 24.9–68.6 years; mean BMI 48.5 kg/m^2^).

Exclusion criteria were smoking, pregnancy, acute or chronic diseases and the use of any medication that could influence energy metabolism or body composition. A total of 3 subjects of the whole study population were over 60 years old; we did not perform cognitive tests performed in this small subgroup of older subjects. Informed written consent to participate in one of the studies was obtained from each subject. All studies were conducted according to the guidelines laid down in the “Declaration of Helsinki” and had been approved by the ethical committee of the department of medicine (Christian-Albrechts University Kiel (A126/09 and A 138/09).

### 2.2. Anthropometric Measurements and Detailed Body Composition Analysis

Body height was measured to the nearest 0.5 cm with subjects wearing no shoes (seca stadiometer; Hamburg, Germany). Weight was assessed to the nearest 0.01 kg with an electronic scale (Tanita, Tokyo, Japan).

Fat mass (FM) and fat free mass (FFM) were assessed by Air Displacement Plethysmography (ADP). ADP was performed by the BOD POD^®^ device (Cosmed s.r.l., Rome, Italy). Participants wore tight-fitting underwear and a swim cap. Two repeated measurements of body volume were performed, averaged and corrected for predicted body surface area and thoracic gas volume using BOD POD^®^ software (version 4.5.0, COSMED srl, Rome, Italy). FM was calculated from body density using the equation by Siri et al. [25] with FFM as bodyweight minus FM. 

Brain volume was assessed by Magnetic resonance imaging (MRI). Scans were obtained using a 1.5T Magnetom Vision scanner (Siemens, Erlangen, Germany). The brain was examined with a T1-weighted sequence (FLASH) (time to repeat: 170.0 ms; time of echo: 4.1 ms/echo). The slice thickness was 6 mm for the brain [26]. As previously described [4] brain scans were segmented into gray matter (GM), white matter (WM) and cerebrospinal fluid (CSF) using statistical parametric mapping (SPM12, http://www.fil.ion.ucl.ac.uk/spm) running on Matlab 2015a (Mathworks, Inc., Natick, MA, USA). 

Intracranial volume (ICV = GM + WM + CSF) was calculated. Additionally, a normalized measure of atrophy, the brain parenchymal fraction volume (BPF), was considered as the relation of brain parenchymal tissue volume (GM plus WM) to ICV. GM and WM masses were calculated using specific densities (GM 1.0385 g/cm^3^; WM 1.0433 g/cm^3^) [27]. As described elsewhere [5,28], the association between brain mass ((B = GM + WM) (g)) and body metabolism ((M) (mlO2/min)) was calculated as the product of subjects individual measured brain mass and 0.0349 mlO2/min/brain mass (g) (cerebral oxygen consumption according to [29]) and the ratio between body and brain metabolism.

### 2.3. Resting Energy Expenditure (REE) Encephalic Measure (EM) 

REE was measured by indirect calorimetry with an open-circuit ventilated-hood system (Vmax Spectra 29n, SensorMedics BV, Viasys Healthcare, Bilthoven, Netherlands; software V-max version 12-1A) after an overnight fast in the early morning; and a detailed protocol was previously reported [18,30]. In our setting, the coefficient of variance for repeated measurements of REE was 5.0% [31]. Gas calibrations of the IC system were performed before and after each measurement period of at least 45 min. We regularly validated our metabolic cart against ethanol combustion. REE was adjusted for FFM (REE_adjFFM_) as previously described [32]. Adaptive thermogenesis (AT) was then calculated from differences in REE_adjFFM_ before and after weight loss [33].

### 2.4. Formatting of Mathematical Components

EM was predicted by the following Equation (1), as previously described [28].

(1) $$Encephalic\;measure=\frac{B_{obs.}}{B_{pred.}}=\frac{B_{obs.}}{B\left(M_{obs.}\right)}=\frac{B_{obs.}}{{M_{obs.}}^{1.03}\times 10^{-0.06}},$$

(2) $$Brain\;predicted=\;{M_{obs.}}^{1.03}\times 10^{-0.06}$$

Encephalic measure (EM) was calculated as a measure of energy distribution from the body (M_obs._) to brain mass (B_obs._ and B_pred._). B_pred._ was calculated as a function of M_obs._ (Equation (2)). Whereas, M_obs._ was calculated as O_2_ consumption in ml/min. based on individual measured REE (indirect calorimetry).

### 2.5. Statistical Analyses

Statistical analyses were performed using SPSS statistical software (SPSS 22.0, Inc., Chicago, IL, USA). All data are presented as mean ± standard deviation (SD) and range (minimal to maximal). Distribution of normality was tested by a Kolmogorov-Smirnov test. Differences among variables between baseline (before weight loss) and follow-up (17.9 ± 7.1 weeks) were analyzed using paired samples *t*-test for normally distributed variables. A general linear model (GLM) with repeated measures was used to test the main effects within and between-subjects, interaction effects between factors, covariate effects and effects of interactions between covariates and between-subject factors. Follow-up time measured in weeks was used as a covariate, two time points of brain imaging were used as the main effect in the model, and weight loss was used as the intervention. Calculation of residuals of REE on FFM was performed by linear regression analysis. Linear regression analysis was used to test the association between age, FM, FFM (minus brain), ICV, GM, WM and energy expenditure variables. Tolerance and variance inflation factor (VIF) were set by <0.01 and >10.0 respectively to indicate multi-co-linearity [34]. Differences in correlation coefficients were tested by using the method of Steiger et al. [35]. A *p*-value < 0.05 was accepted as the limit of significance.

## 3. Results

### 3.1. Is There an Effect of Weight Loss on Brain Mass, GM, WM and REE?

Data on body composition, brain compartments and energy expenditure are presented in Table 1. There were significant changes in weight (−14.5 ± 11.9 kg), total FM (−12.9 kg ± 9.8 kg), and FFM (−1.7 ± 4.8 kg) (*p* < 0.05). Both, REE (−159 ± 191 kcal/24 h) and REE_adjFFM_ (−159 ± 177 kcal/24 h) decreased with weight loss (*p* < 0.05). While there were no differences in absolute GM, WM and BPF, weight change was associated with changes in CSF (9.04 mL ± 20.16), ICV (13.93 mL ± 55.34), EM (0.43 ± 0.50) and the ratio between body metabolism and brain metabolism (−0.63 ± 0.76; *p* < 0.05).

The general linear model (GLM) with repeated measures showed that within subjects an intervention had a significant (*p* < 0.05) effect on ICV changes and 6.9% of these changes could be explained by the intervention, but not by the time of intervention. As to GM changes within subjects, the intervention showed no effect, but an interaction of time x intervention explained 6.0% (*p* < 0.05). In addition, between-subjects 12.8% of the variance in GM changes could be explained by time, i.e., they increased with the duration of observation time. The intervention explained 20.1% of the variance in changes of CSF. Between-subjects time explained 11.6% of the variance in CSF changes.

**Figure 1:**
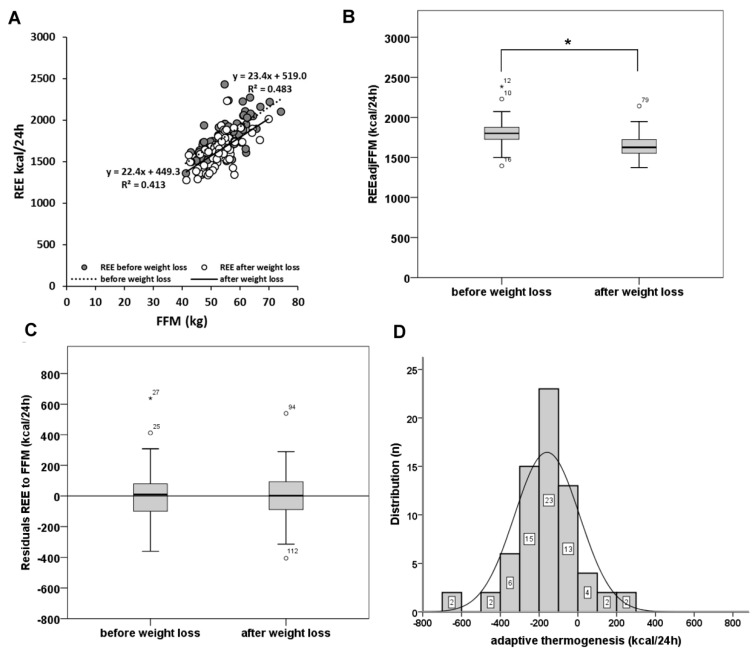
Relationship (**A**) between resting energy expenditure (REE (kcal/24 h)) and fat free mass (FFM (kg)) before and after weight loss, (**B**) resting energy expenditure (REE (kcal/24 h)) adjusted for fat free mass (FFM (kg)) before and after weight loss resting and (**C**) energy expenditure (REE kcal/24 h) and fat free mass (FFM (kg)) residuals before and after weight loss. (**D**) Distribution of adaptive thermogenesis in the study population. * Significant different by paired *t*-test (*p* < 0.05).

Weight loss resulted in a decrease in REE (Table 1) and REE_adjFFM_ (Table 1, Figure 1B). REE was closely related to FFM (Figure 1A). However, weight loss did not affect their relationship. There was no significant difference in REE on FFM residuals before and after weight loss (Figure 1C). Adaptive thermogenesis with weight loss ranged between −672 and 294 kcal/24 h (Figure 1D).

### 3.2. Contribution of GM and WM to the Variances in REE, REE_adjFFM_ and REE on FFM Residuals before and after Weight Loss

There was a positive correlation between WM and REE (*r* = 0.353; *p* < 0.05) before weight loss. By contrast, GM correlated with the REE on FFM residuals (*r* = 0.237; *p* < 0.05) after weight loss only.

Different multiple stepwise linear regression analyses were made to analyze if a single brain compartment added to variances in REE, REE_adjFFM_, and REE on FFM residuals and adaptive thermogenesis (Table 2). While ICV did not add to the variances in REE, REE_adjFFM_ and REE on FFM residuals after weight loss, GM added 3.6% (ß = 0.190; *p* = 0.022), 6.1% (ß = 0.248; *p* = 0.026) and 6.6% (ß = 0.252; *p* = 0.021) to the explained variances in REE, REE_adjFFM_ and REE on FFM residuals after weight loss. There were no associations between either ICV or GM or WM and adaptive thermogenesis.

**Figure 2:**
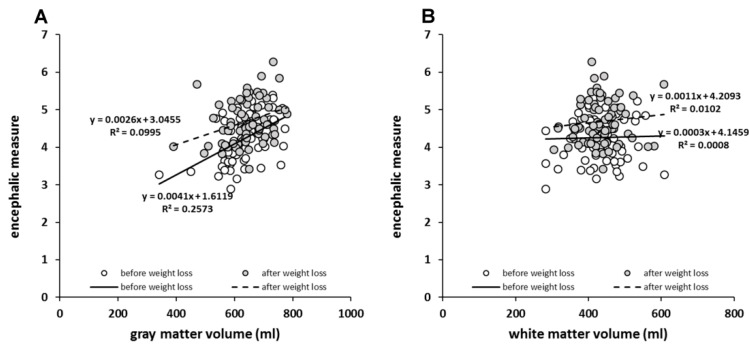
Relationship between encephalic measure and volumes of (**A**) gray matter and (**B**) white matter before and after weight loss.

The volume of GM showed significant (*p* < 0.05) and positive associations with EM before (*r* = 0.507) and after weight loss (*r* = 0.316) (Figure 2A). This was not true in WM volume (Figure 2B).

The variance of changes in EM was mainly explained by GM volume (*r*^2^ = 0.068; ß = 0.261; *p* = 0.030). Concomitantly, there was no relationship between changes in EM and WM.

## 4. Discussion

### 4.1. Is There an Effect of Weight Loss on Brain Mass and Brain Tissue Structure?

In obese patients weight loss did not affect absolute brain mass, GM and WM (Table 1 and Figure 1). By contrast, CSF, ICV, EM and the ratio between body and brain metabolism increased with weight loss (Table 1). While there was no effect of weight loss on changes in metabolically active brain compartments (GM and WM), the body-brain energy allocation increased with weight loss and GM and WM added to the variances of REE and REE on FFM residuals.

With weight loss, there was a considerable increase in EM (Table 1). EM reflects energy allocation between the brain and all other organs and tissues of the body. We have previously shown that, when compared to a normal weight women EM was low in subjects with obesity, while EM improved with weight loss [28]. Metabolism of body and brain are related in an “isometric” way, i.e., REE decreased with weight loss; whereas, brain energy metabolism remained stable. Thus, with weight loss, the ratio between body to brain metabolism decreased from 6.68 ± 0.99 to 6.05 ± 0.72 towards an ideal ratio of 5 [6]. A ratio of 5 reflects a brain energy consumption of approximately 20% of REE. In our study population, brain energy consumption increased from 15.3% to 16.8% of body metabolism, and thus, was lower than 20%. Our data are in line with earlier data showing that subjects with obesity had lower brain energy consumption (17.2 ± 0.1%) than normal weight subjects [6].

GM was positively associated with EM before and after weight loss. A higher GM volume was related to an increased EM indicating that more energy is allocated from the body to GM. Furthermore, GM contributed to the explained variances of REE, REE_adjFFM_ and REE on FFM residuals after weight loss only. The present longitudinal data fits our previous findings based on cross-sectional data [4]. When compared to WM, GM had a higher specific metabolic rate and had a stronger relation to EM which could confirm the assumption that after weight loss the body-brain- (i.e., body-GM-) energy allocation increased.

In conclusion, weight loss had no effect on the volumes of metabolically active brain compartments (GM and WM). After weight loss, GM added to the variances of REE and REE on FFM residuals, and influenced the body-brain energy allocation. Our data are in line with more recent data obtained in obese patients before and after two years after bariatric surgery [36]. The results showed no impact of weight loss on adjusted REE and brain mass [36].

### 4.2. Strengths and Limitations

Strength of the presented study is that we could use detailed body composition data assessed by MRI in a longitudinal data set of females. Moreover, we have assessed brain tissue composition, GM, WM and CSF. Limitations were the limited quality of MRI brain scans obtained using a 1.5T scanner only. Thus, it was not possible to assess changes in individual brain regions, as well as their corresponding densities. In addition, we did not assess potential obesity-related pro-inflammatory proteins, metabolic disruptions or lipotoxicity, which could influence diverse brain structures [37,38,39,40]. It is possible that there are changes in deeper structures (e.g., density of specific brain regions) of GM and WM, e.g., metabolism of brain structures is directly related to the functionality of neuronal density and brain volumes, with reduced neuronal densities, but larger neurons, may show decreased specific metabolic rates [41].

## Figures and Tables

**Table 1:** Body composition and energy expenditure characteristics before and after weight loss (females; *n* = 69).

	*n*	Before Weight Loss			After Weight Loss			Δ
		Mean ± SD	Minimal	Maximal	Mean ± SD	Minimal	Maximal	Mean ± SD
Age (years)	69	36.4 ± 9.4	19.4	68.8	36.72 ± 9.9	19.6	69.4	
Height (m)	69	1.68 ± 0.07	1.49	1.82	1.68 ± 0.07	1.49	1.83	0.00 ± 0.00
Weight (kg)	69	110.3 ± 23.2	72.6	159.0	95.7 ± 17.5	67.1	135.5	−14.5 ± 11.9 *
BMI (kg/m^2^)	69	38.9 ± 7.5	28.2	58.7	33.8 ± 5.0	25.4	44.5	−5.2 ± 4.3 *
Body composition								
FM (%)	69	49.2 ± 6.8	32.6	68.8	43.5 ± 7.4	19.80	56.4	−5.7 ± 4.2 *
FM (kg)	69	55.5 ± 18.2	26.2	104.7	42.7 ± 13.9	14.2	70.4	−12.9 ± 9.8 *
FFM (kg)	69	54.68 ± 7.01	41.2	74.1	52.9 ± 5.5	41.4	69.7	−1.7 ± 4.8 *
Gray matter (mL)	69	638.9 ± 74.7	340.9	773.1	638.3 ± 71.8	390.6	779.3	−0.6 ± 36.9
Gray matter (ratio to ICV)	69	0.50 ± 0.04	0.31	0.58	0.49 ± 0.04	0.35	0.59	−0.005 ± 0.03
White matter (mL)	69	430.3 ± 65.6	282.2	608.6	435.9 ± 53.9	305.2	606.8	5.5 ± 43.3
White matter (ratio to ICV)	69	0.34 ± 0.05	0.24	0.55	0.34 ± 0.04	0.25	0.51	0.001 ± 0.03
Cerebrospinal Fluid (mL)	69	202.1 ± 54.6	115.4	402.4	211.2 ± 54.3	119.3	408.6	9.0 ± 20.2 *
Cerebrospinal Fluid (ratio to ICV)	69	0.16 ± 0.04	0.09	0.29	0.16 ± 0.04	0.10	0.29	0.004 ± 0.02 *
Intracranial Volume (mL)	69	1271.4 ± 94.1	1099.7	1477.7	1285.3 ± 97.4	1100.4	1485.6	13.9 ± 55.3 *
Brain parenchymal fraction (mL)	69	1069.3 ± 92.0	868.6	1241.5	1074.2 ± 80.5	915.0	1241.4	4.9 ± 53.1
Brain parenchymal fraction (ratio to ICV)	69	0.84 ± 0.04	0.71	0.91	0.84 ± 0.04	0.71	0.90	−0.004 ± 0.01 *
Encephalic measure	69	4.25 ± 0.61	2.90	5.40	4.68 ± 0.58	3.42	6.29	0.43 ± 0.50 *
Ratio of body metabolism to brain metabolism	69	6.68 ± 0.99	5.18	9.56	6.05 ± 0.72	4.47	8.10	−0.63 ± 0.76 *
Energy expenditure								
REE (kcal/24 h)	69	1799 ± 236	1346	2433	1640 ± 191	1282	2234	−159 ± 191 *
REE_adjFFM_ (kcal/24 h)	69	1799 ± 169	1082	3070	1640 ± 146	942	2773	−159 ± 177 *
REE on FFM residuals	69	0.00 ± 169	−360	638	0.00 ± 146	−405	540	0.00 ± 177

Data are presented as means ± standard deviation (Mean ± SD); Abbreviations: Δ difference after minus before weight loss; BMI, body mass index; FFM, fat free mass; FM, fat mass; REE, resting energy expenditure; REE_adjFFM_, resting energy expenditure adjusted for fat free mass; ICV, Intracranial Volume (Sum of gray matter, white matter and cerebrospinal fluid). Significant differences before and after weight loss are indicated by * as tested with paired *t*-test (*p* < 0.05).

**Table 2:** Multivariate regression analysis (stepwise) with energy expenditure as the dependent variable.

	Regression Coefficient B	SE	ß-Coefficient	*p*-Value	Regression Coefficient B	SE	ß-Coefficient	*p*-Value
	REE Before weight loss				REE after weight loss			
Model 1								
Constant	545.9	293.8		0.068	520.29	260.3		0.050
FFM (kg)	14.13	3.39	0.419	0.001	14.27	3.48	0.407	0.001
FM (kg)	5.83	1.31	0.449	0.001	5.94	1.38	0.433	0.001
Age	−1.04	2.02	−0.043	0.611	−1.75	1.67	−0.091	0.296
ICV (mL)	0.17	0.20	0.066	0.419	0.14	0.17	0.075	0.377
R² total	0.611				0.550			
Model 2								
Constant	720.9	146.6		0.001	274.9	211.3		0.198
FFM (kg)	14.15	3.33	0.420	0.001	15.37	3.32	0.438	0.001
FM (kg)	5.76	1.28	0.444	0.001	5.73	1.29	0.417	0.001
Age								
GM (mL)					487.4	208.2	0.190	0.022
WM (mL)								
R² total	0.603				0.574			
	REE_adjFFM_ before weight loss				REE_adjFFM_ after weight loss			
Model 3								
Constant	1515.5	289.1		0.001	1351.6	277.5		0.001
FM (kg)	3.54	1.05	0.379	0.001	4.23	1.22	0.402	0.001
Age	−1.68	2.10	−0.098	0.427	−1.14	1.71	−0.077	0.508
ICV (mL)	0.09	0.212	0.048	0.628	0.097	0.171	0.065	0.572
R² total	0.157				0.166			
Model 4								
Constant	1576.8	61.6		0.001	1109.3	153.2		0.001
FM (kg)	3.54	1.05	0.379	0.001	4.28	1.15	0.407	0.001
Age								
GM (mL)					487.3	214.0	0.248	0.026
WM (mL)								
R² total	0.144				0.217			
	REE on FFM residuals before weight loss				REE on FFM residuals after weight loss			
Model 3								
Constant	−227.6	289.0		0.340	−218.5	227.4		0.220
FM (kg)	3.55	1.05	0.380	0.001	4.24	1.22	0.403	0.001
Age	−1.77	2.10	−0.103	0.402	−1.22	1.71	−0.082	0.479
ICV (mL)	0.11	0.21	0.058	0.623	0.113	0.17	0.075	0.512
R² total	0.160				0.168			
Model 4								
Constant	−197.2	67.7		0.002	−518.9	152.9		0.001
FM (kg)	3.55	1.05	0.380	0.001	4.29	1.14	0.408	0.001
Age								
GM (mL)					506.4	213.7	0.258	0.021
WM (mL)								
R² total	0.163				0.222			

Model 1 included: FFM (kg), FM (kg), Age, ICV (mL); Model 2 included: FFM (kg), FM (kg), Age, GM (mL) and WM (mL); Model 3 included: FM (kg), Age, ICV (mL); Model 4 included: FM (kg), Age, GM (mL) and WM (mL). Abbreviations: FFM, fat free mass; FM, fat mass; ICV, intracranial volume; GM, gray matter; WM, white matter; REE, resting energy expenditure; REE_adjFFM_, resting energy expenditure adjusted for fat free mass; SE, standard error.
